# Demonstrating a reduced capacity for removal of fluid from cerebral white matter and hypoxia in areas of white matter hyperintensity associated with age and dementia

**DOI:** 10.1186/s40478-020-01009-1

**Published:** 2020-08-08

**Authors:** Matthew MacGregor Sharp, Satoshi Saito, Abby Keable, Maureen Gatherer, Roxana Aldea, Nivedita Agarwal, Julie E. Simpson, Stephen B. Wharton, Roy O. Weller, Roxana O. Carare

**Affiliations:** 1 Faculty of Medicine, University of Southampton, Tremona Road, Southampton, SO16 6YD UK; 2 Roche Pharma Research and Early Development (pRED), Pharmaceutical Sciences, Roche Innovation Center Basel, Basel, Switzerland; 3 Hospital Santa Maria del Carmine, Rovereto, Italy; 4 Sheffield Institute for Translational Neurosciences, University of Sheffield, Sheffield, UK

**Keywords:** White matter hyperintensities, Laminin, Fibronectin, Intramural periarterial drainage

## Abstract

White matter hyperintensities (WMH) occur in association with dementia but the aetiology is unclear. Here we test the hypothesis that there is a combination of impaired elimination of interstitial fluid from the white matter together with a degree of hypoxia in WMH. One of the mechanisms for the elimination of amyloid-β (Aβ) from the brain is along the basement membranes in the walls of capillaries and arteries (Intramural Peri-Arterial Drainage – IPAD). We compared the dynamics of IPAD in the grey matter of the hippocampus and in the white matter of the corpus callosum in 10 week old C57/B16 mice by injecting soluble Aβ as a tracer. The dynamics of IPAD in the white matter were significantly slower compared with the grey matter and this was associated with a lower density of capillaries in the white matter. Exposing cultures of smooth muscle cells to hypercapnia as a model of cerebral hypoperfusion resulted in a reduction in fibronectin and an increase in laminin in the extracellular matrix. Similar changes were detected in the white matter in human WMH suggesting that hypercapnia/hypoxia may play a role in WMH. Employing therapies to enhance both IPAD and blood flow in the white matter may reduce WMH in patients with dementia.

## Introduction

White Matter Hyperintensities (WMH) on magnetic resonance imaging (MRI) occur in the cerebral hemispheres mainly in elderly patients and are particularly associated with dementia [1]. Hyperintensity on MRI suggests that there is fluid in the white matter and is typically referred to as leukoaraiosis on CT [2, 3]. Various mechanisms for the aetiology of WMH have been proposed, they include ischaemia/hypoxia as a result of arteriosclerotic small vessel disease [1] loss of axons in the white matter associated with deposition of tau protein in parent neurons in the overlying grey matter [2] and a failure of elimination of interstitial fluid (ISF) from the affected white matter along peri-capillary and peri-arterial drainage routes [4].

The brain has no conventional lymphatic vessels. Early physiological studies using radio-iodinated tracers have shown that fluid, soluble metabolites and tracers drain along the walls of cerebral arteries to cervical lymph nodes and only an estimated 10–15% leaks into the CSF [5, 6]. Detailed anatomical studies of this lymphatic drainage pathway have shown that soluble tracers, including soluble amyloid-β (Aβ), drain from the extracellular spaces of the brain along basement membranes (BM) in the walls of cerebral capillaries and then continue out of the brain along BMs that surround smooth muscle cells in the tunica media of arteries [7]. This drainage pathway is termed the Intramural Peri-Arterial Drainage (IPAD) pathway [8]. Aβ in the extracellular spaces of the brain is taken up by perivascular macrophages, micoglia and astrocytes, crosses into the blood via low density lipoprotein related protein − 1 (LRP1) and also enters the IPAD pathways [9]. IPAD becomes less efficient with advancing age, leading to the accumulation of insoluble Aβ in the BMs of the IPAD pathways as cerebral amyloid angiopathy (CAA) [10, 11]. The elimination of soluble Aβ is severely impaired in Alzheimer’s disease (AD) with a rise in levels of soluble Aβ in the brain parenchyma suggesting that a loss of homeostasis in the extracellular spaces of the brain accompanies dementia in AD [12].

It appears, therefore, that two of the major functions of arteries, arterioles and capillaries in the brain are (a) maintaining blood flow with the supply of nutrients and immune cells to brain tissues and (b) drainage of interstitial fluid and soluble metabolites from the brain to cervical lymph nodes by the IPAD pathways to maintain homeostasis in the brain [13, 14]. Changes that occur in cerebral arteries and arterioles associated with age, such as arterioslerosis and CAA, affect both blood flow and IPAD and may induce both ischaemia/hypoxia and loss of homeostasis due to impaired elimination of ISF and soluble metabolites from the brain along IPAD pathways.

The process of IPAD ceases upon cardiac arrest [7], therefore it was initially assumed that IPAD was driven by the pulsation force of cerebral vessels [15]. However, mathematical simulations by Diem et al. confirmed that this would not provide sufficient motive force [16]. More recently, new mathematical models and in-vivo 2 photon microscopy on awake mice suggest that it is vasomotion generated by cycles of contraction and relaxation of smooth muscle cells that drives IPAD. Vasomotion induces deformations of the BM, effectively opening and closing a valve like system allowing for flow of IPAD in the direction of the vasomotion wave [17, 18]. Motive force of vascular smooth muscle cells decreases with age and in CAA, resulting in hypoperfusion, ischaemia and a failure of IPAD [17, 19, 20].

Cerebrovascular BM consist of a fine extracellular matrix (ECM) of glycoproteins and proteoglycans that ensheath the abluminal side of endothelia separating the endothelia from pericytes or smooth muscle cells and pericytes or smooth muscle cells from astrocytes, encircling the different cell types [21]. Each cell type contributes to ECM production. The ECM consists of highly crosslinked complexes of collagen IV, laminin, nidogen/entactin, fibronectin and heparan sulphate proteoglycan. Remodelling of the ECM is a common feature in ageing and neuropathological conditions [22, 23]. Experimental work in rodents demonstrates that ischaemia in the white matter is characterised by an upregulation of the extracellular matrix proteins laminin and fibronectin [24–26] but it is not known if similar changes occur in human white matter. As BMs are key to IPAD, remodelling of the ECM associated with ischaemia will likely have an impact on the IPAD pathways.

In this study we aim to firstly ascertain if ISF and solutes are eliminated from the white matter by IPAD. We use young adult wild-type mice to compare the drainage of amyloid-β (1–40) from hippocampal grey matter and the white matter tracts of the corpus callosum. We then use human post-mortem brains with WMH to compare the levels of extracellular matrix proteins fibronectin and laminin with those of age-matched controls. Finally, we apply hypercapnia to model hypoxic hypoperfusion and analyse the production of fibronectin and laminin by cultured human brain vascular smooth muscle cells (HBVSMC). Involvement of failure of IPAD in the aetiology of WMH has previously been deduced largely from circumstantial evidence and involvement by association. Direct evidence is difficult to obtain, especially from post-mortem human brain tissue. In this study we seek more direct evidence for the involvement of failure of IPAD in the aetiology of WMH. We show evidence of hypoxia in the white matter in WMH and we also demonstrate, experimentally, a reduced capacity of IPAD in the white matter compared with grey matter.

We test two hypotheses: *Hypothesis 1:* The dynamics of IPAD in the cerebral white matter differ from IPAD in the grey matter of the hippocampus. The hypothesis is tested by a) comparing the density of capillaries in grey and white matter and b) injecting soluble Aβ as a tracer independently into the grey matter of the hippocampus and into the white matter of the corpus callosum of mice and comparing the dynamics of drainage of tracer along IPAD from each of these regions of the brain. *Hypothesis 2:* Changes induced by hypercapnia as a model of hypoxia in the extracellular matrix of vascular smooth muscle cells are similarly expressed in human white matter exhibiting WMH. In order to test Hypothesis 2 we selected two proteins in the BMs of smooth muscle cells, a) laminin and b) fibronectin and established the effects of hypercapnia as a model of hypoxiaon cultures of vascular smooth muscle cells. We then compared the changes observed in culture with the changes in extracellular matrix in WMH.

## Materials and methods

### Stereotaxic injections of amyloid-β (1–40) HiLyte Fluor 555 into mouse hippocampus (grey matter) and corpus callosum (white matter) and quantification of IPAD

All procedures were carried out in accordance with animal care guidelines stipulated by the United Kingdom Animals (Scientific Procedures) Act 1986, Home Office licence P12102B2A. 10-week-old C57/BL6 wild-type mice (*n* = 10) were anaesthetised with Isoflurane mixed with concentrated O_2_ (1.7 L min-1) (induced with 3%, maintained using 2%). Isoflurane was used rather than injectable anaesthetics based on our previous study which showed that isoflurane is better at maintaining a more physiologically normal heart rate and oxygen saturation level [8]. The level of anaesthesia was monitored by using pedal withdrawal reflex response. A rectal probe and homoeothermic blanket and temperature control system (BASi) were used to regulate internal body temperature at 37 °C. Lacri-lube ointment was applied to the eyes to preserve cornea during anaesthesia.

**Fig. 1:**
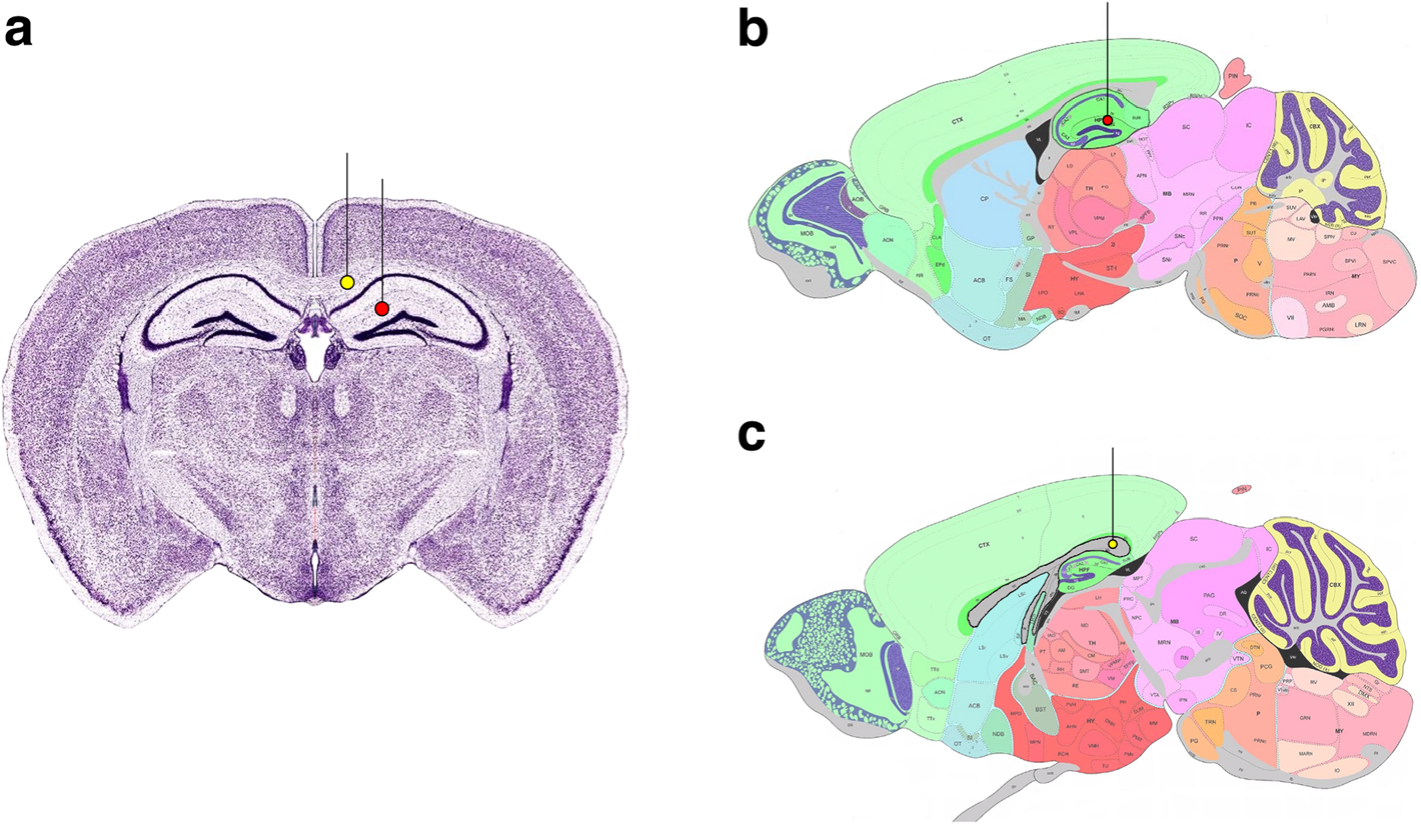
Stereotaxic Injection sites. Injections were performed Anterior-Posterior − 2 mm from the Bregma represented by the red dot (hippocampus) and yellow dot (corpus callosum) in **(a)**. Sagittal slices show the injection site for the hippocampus (Anterior-Posterior - 2 mm; Medial-Lateral 1.5 mm; Dorsal-Ventral 1.7 mm), red dot in **(b)** and corpus callosum (Anterior-Posterior -2 mm; Medial-Lateral 0.5 mm; Dorsal-Ventral - 1.3 mm), yellow dot in **(c)**. Images adapted from www.mouse.brain-map.org

Anaesthetised mice were placed in a KOPH instruments stereotaxic frame (Model 900) and the head secured with jaw bars. A midline incision was performed and a Tech2000 Micromotor drill (RAM Products, INC) with 0.7 mm burr was used to create a burr hole in the skull above the injection site (hippocampal grey matter - Anterior-Posterior - 2 mm; Medial-Lateral 1.5 mm; Dorsal-Ventral - 1.7 mm, *n* = 5 or corpus callosum (white matter) - Anterior-Posterior − 2 mm; Medial-Lateral 0.5 mm; Dorsal-Ventral - 1.3 mm, *n* = 5) (Fig. 1). 0.5 μl of 100 μM amyloid-β (1–40) HiLyte Fluor 555 (Cambridge Bioscience) was injected into either the hippocampus or corpus callosum using a Hamilton Neuros Syringe with a 33 gauge needle (Essex Scientific Laboratory Supplies Ltd.) and Microinjection syringe pump (UMP3T-1; World Precision Instruments) at a rate of 0.25 μl min-1. The syringe was left in situ for 2 min for bolus diffusion and to prevent reflux. The tracers were left to drain for a further 5 min and then the mouse was terminally anaesthetised with pentobarbitone (200 mg/kg) and intracardially perfused with 0.01 M phosphate buffered saline (PBS) followed by 4% Paraformaldehyde (PFA) in 0.01 M PBS, pH 7.4 at a rate of 5 ml/min. Brains were dissected and post fixed for 6 h in fresh 4% PFA in 0.01 M PBS, pH 7.4 at 4 °C and then cryoprotected in 30% sucrose in distilled H_2_O at 4 °C for a further 48 h. Brains were embedded in OCT compound and then sectioned into 20 μm coronal slices using a Leica CM1860 UV cryostat. Sections were collected on to SuperFrost Plus™ adhesion slides (Thermo Scientific™, 10,149,870) and viewed using a Zeiss Axioskop 2 fitted with a rhodamine filter to identify the section containing the site of injection. In our previous studies we showed that, in mice, the drainage of Aβ40 occurs predominantly in a posterior direction and can be visualised in the walls of blood vessels as close as 200 μm to the injection site [10]. We therefore chose coronal sections 200 μm posterior to the injection site for immunohistochemistry.

Sections were washed 2 × 3 mins in 0.01 M PBS, pH 7.4 and then blocked in 15% goat serum (Sigma 9023) for 1 h at room temperature. Sections were then incubated in rabbit anti-collagen IV 1/400 in 0.01 M PBSt (AbCam, ab6586) and anti-smooth muscle actin (SMA) FITC conjugated 1/200 in 0.01 M PBSt (Sigma, F3777) overnight in a moist chamber at 4 °C. Sections were then washed 3 × 10 mins in 0.01 M PBS and incubated in conjugated secondary antibody goat antirabbit Alexa Fluor 633 0.01 M PBSt (ThermoFisher Scientific, A-21070) for 1 h at room temperature. Sections were further incubated in 1% Sudan Black for 5 min to remove auto fluorescence before being mounted in Mowiol Citifluor and imaged using confocal microscopy.

For each section, tile scans of the left hippocampus or corpus callosum were captured using a Leica SP8 confocal microscope fitted with × 20 objective set at an optical zoom of 1. Laser power and detection windows were kept consistent for all scans. Sequential imaging was used to prevent cross excitation of fluorophores. Quantification of IPAD of Aβ40 HiLyte Fluor 555 injected into the left hippocampus or corpus callosum was performed using a max projection of each tile scan uploaded into Adobe Photoshop CS6. We chose to use maximum projections of each tile scan as they provide a more precise and accurate method to assess IPAD, particularly when assessing vessel density. IPAD was assessed by using Adobe Photoshop CS6 to manually measure vessel density of capillaries, arterioles and venules and counting the number of vessels containing Aβ40 HiLyte Fluor 555 in their vessel walls. Vessel density was calculated by dividing the total number of capillaries, arterioles or venules by the overall surface area (in μm^2^) and multiplying this value by 500,000 to be expressed as number of vessels per 0.5mm^2^. This was also performed for the number of capillaries, arterioles or venules with Aβ40 in their vessel walls. Vessels were identified based on lumen diameter and immunoreactivity to SMA (< 10 μm = capillaries, ≥ 10 μm & SMA positive = arterioles, ≥ 10 μm and SMA negative = venules) [7, 10]. The overall surface area was determined by choosing regions of interest that were based on key anatomical features that could be observed in each section analysed. Regions of interest were outlined using Adobe Photoshop CS6. For the hippocampus, a transverse area from the edge of the suprapyramidal [16] was outlined. For the corpus callosum, the white matter tracts extending from the midline to a point directly above the suprapyramidal blade of the granule cell layer of the hippocampus were outlined. Statistical analysis was performed using SPSS Statistics version 26.0 (IBM) and an independent t-test with significance set at *P* < 0.05.

### Assessment of fibronectin and laminin expression in WMH

Human autopsy CNS tissue was obtained from the MRC Cognitive Function and Ageing Study (CFAS) that had a post-mortem MRI confirming white matter hyperintensities [27, 28]. Control cases were from CFAS and Parkinson’s UK brain bank. The Parkinson’s UK Brain Bank is funded by Parkinson’s UK, a charity registered in England and Wales (258197) and in Scotland (SC037554) (Table 1).

**Table 1:** Demographics of cases used in this study. pm delay = postmortem delay in hours

Source	Age	Sex	pm delay /hrs	Category
CFAS	87	F		White matter hyperintensity
CFAS	84	F		White matter hyperintensity
CFAS	78	F		White matter hyperintensity
CFAS	91	F		White matter hyperintensity
CFAS	95	F		White matter hyperintensity
CFAS	91	F		White matter hyperintensity
CFAS	89	M		White matter hyperintensity
CFAS	83	M		White matter hyperintensity
CFAS	73	M		White matter hyperintensity
CFAS	88	F		White matter hyperintensity
CFAS	76	M		Control
CFAS	88	M		Control
CFAS	89	F		Control
CFAS	91	M		Control
Parkinson’s UK	77	M	17	Control
Parkinson’s UK	90	M	12	Control
Parkinson’s UK	93	F	36	Control
Parkinson’s UK	92	F	24	Control
Parkinson’s UK	96	F	24	Control
Parkinson’s UK	85	M	29	Control
Parkinson’s UK	79	M	25	Control

Two tissue sections of 10 μm thickness of the white matter from each case (WMH × 10, Control × 11) were deparaffinised and then rehydrated through a graded series of alcohols. Endogenous peroxidase activity was quenched with 3% hydrogen peroxide for 15 min at room temperature. Heat mediated antigen retrieval was then performed by microwaving in 0.01 M citrate buffer (pH 6). After blocking in 15% goat serum (Sigma, 9023) for 1 h at room temperature, sections were incubated in rabbit polyclonal anti-laminin antibody 1/50 (Merck, L9393) or rabbit polyclonal anti-fibronectin antibody 1/400 (Merck,) in 0.01 M PBS with 0.1% triton in a moist chamber at 4 °C for 48 h. Sections were then washed 3 × 10 mins in 0.01 M PBS and incubated in goat anti-rabbit IgG 1:200 (Vector Laboratories) in 0.01 M PBS with 0.1% triton for 1 h at room temperature. Sections were further incubated with the alkaline phosphatase substrate (Vector Laboratories, PK-6101) for 1 h at room temperature. Anti-laminin or anti-fibronectin immunoreactivity was visualised by incubating tissue sections in glucose oxidase diaminobenzidine nickel (DAB) solution for 5 mins (Vector Laboratories, SK-4100). Sections were then washed, dehydrated, cleared in xylene and coverslipped with DPX (Thermo Scientific).

Tissue sections were examined using an Olympus dot slide microscope. Randomly selected regions (1.7 mm × 2.2 mm) of white matter (1 per slide) were captured from each section and imported into Image J software (National Institutes of Health) [29] to calculate total area stained for either laminin or fibronectin. To account for possible variations in vessel density, the total area of either laminin or fibronectin staining was normalised against the number of vessels in each region. Statistical analysis was performed using SPSS Statistics version 26.0 (IBM) and Student’s t test with significance set at *P* < 0.05.

### Analysis of fibronectin and laminin production by human brain vascular smooth muscle cells exposed to increased CO_2_ levels

Human brain vascular smooth muscle cells (HBVSMC) were obtained from Sciencell (sc-1100). Cells were grown in the smooth muscle cell medium purchased from Sciencell (sc-1101) supplemented with smooth muscle cell growth supplement (sc-1152), 100 U/mL penicillin, 100 μg/mL streptomycin (sc-0503) and 2% foetal bovine serum. Cells were maintained in a humidified atmosphere (5% CO_2_/95% air) at 37 °C and the medium was refreshed every 2–3 days according to the manufacturer’s instructions. To confirm that the cells are indeed smooth muscle cells, we analysed the presence of smooth muscle actin through immunofluorescence with anti-alpha smooth muscle actin 1/200 in 0.01 M PBSt (Sigma, F3777).

We first assessed for cell proliferation and metabolic activity of HBVSMC in either a normoxic environment or conditions of increased CO_2_ using a CellTiter 96 aqueous one solution cell proliferation assay kit (Promega, G3582). Cells were seeded into two 96 well plates with poly-L-lysine coated wells at a density of 10^4^ cells per well. Plates were incubated for 72 h in either normoxic conditions (5% CO_2_, 95% air) or conditions of increased CO_2_ (8% CO_2_, 92% air) and maintained at 37 °C. 20 μL of MTS reagent was added to the cells and the plates were wrapped in foil and incubated for a further 2 h for the reagent to develop. Blank wells containing no cells were also included and their absorbance value was subtracted from the test wells. Absorbance was measured at 490 nm with a Fluorostar optima plate reader and the results were exported to Microsoft Excel.

We next assessed for expression of laminin or fibronectin from HBVSMC in normoxic and conditions of increased CO_2_. HBVSMC were plated onto poly-L-lysine coated coverslips in a 24-well plate at a density of 0.5 × 10^5^ cells per coverslip with 1 mL of smooth muscle medium. Cells were then exposed to either normoxic (5% CO2/95% air) or conditions of increased CO_2_ (8% CO_2_, 92% air) for 72 h and fixed with 4% paraformaldehyde for 10 min before immunofluorescent staining with either with anti-laminin (Sigma, L9393) or anti-fibronectin (Sigma, F3648) antibodies, diluted to 1:200 and 1:400 in 0.01 M PBS respectively. Cells were examined by using a Leica SP8 confocal microscope and three non-overlapping z-stacks were captured per stained coverslip (each an area of 0.15 mm^2^). The fluorescence intensity of laminin and fibronectin staining was calculated using the RGB measure tool in ImageJ. The results from individual images were averaged to give a mean value per coverslip and are based upon data gathered over three independent experimental runs (*n* = 3). Statistical analysis was performed using SPSS Statistics version 26.0 (IBM) and a Mann-Whitney U test with significant set at *P* ≤ 0.05.

## Results

### ISF and solutes are drained from the white matter by IPAD

To determine the pattern of intramural Peri-Arterial Drainage (IPAD) in the white matter, the pattern of drainage of fluorescent Aβ40 injected into the corpus callosum was compared with the drainage of Aβ40 injected into the hippocampus of young adult (10-week-old) C57/Bl6 mice. Immunohistochemistry and confocal microscopy was used to assess differences / similarities in vascular density and IPAD between the hippocampus and corpus callosum.

**Fig. 2:**
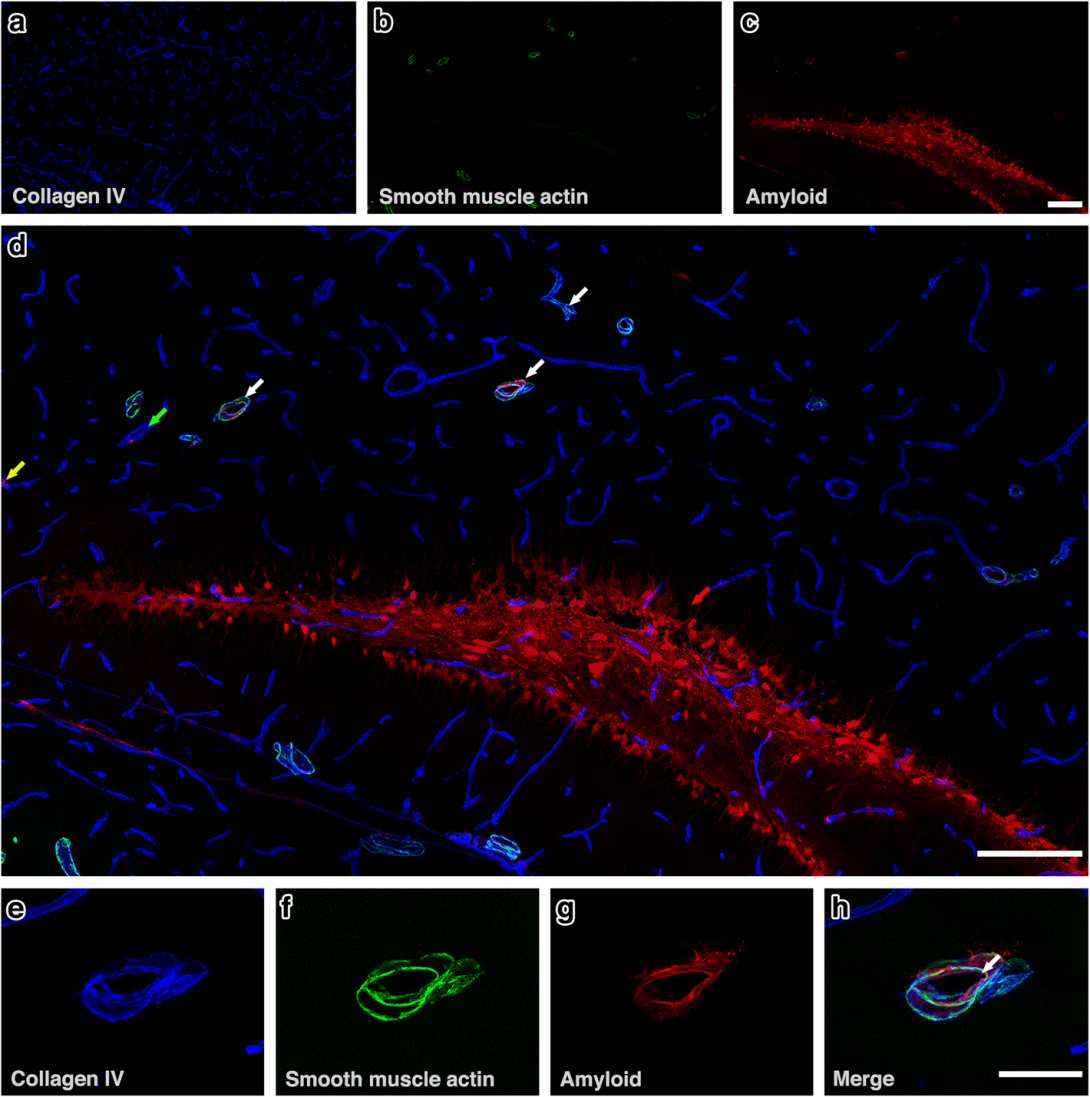
IPAD in hippocampus. **(a -c)** The distribution of amyloid-β in relation to collagen IV and smooth muscle actin at 7 min after intrahippocampal injection. **c & d)** Amyloid-β **(red)** was observed diffusely distributed in the parenchyma and co-localised **(pink colour)** with collagen IV in the walls of arterioles **(white arrows)**, capillaries **(yellow arrow)** and few venules **(green arrow)**. Representative high power image of an arteriole **(e-h)** shows amyloid-β **(red) (g-h)** in the wall of the blood vessel, indicated by the white arrow in **(h)**. Scale bars **a-d** = 200 μm, **e – h** = 10 μm

**Fig. 3:**
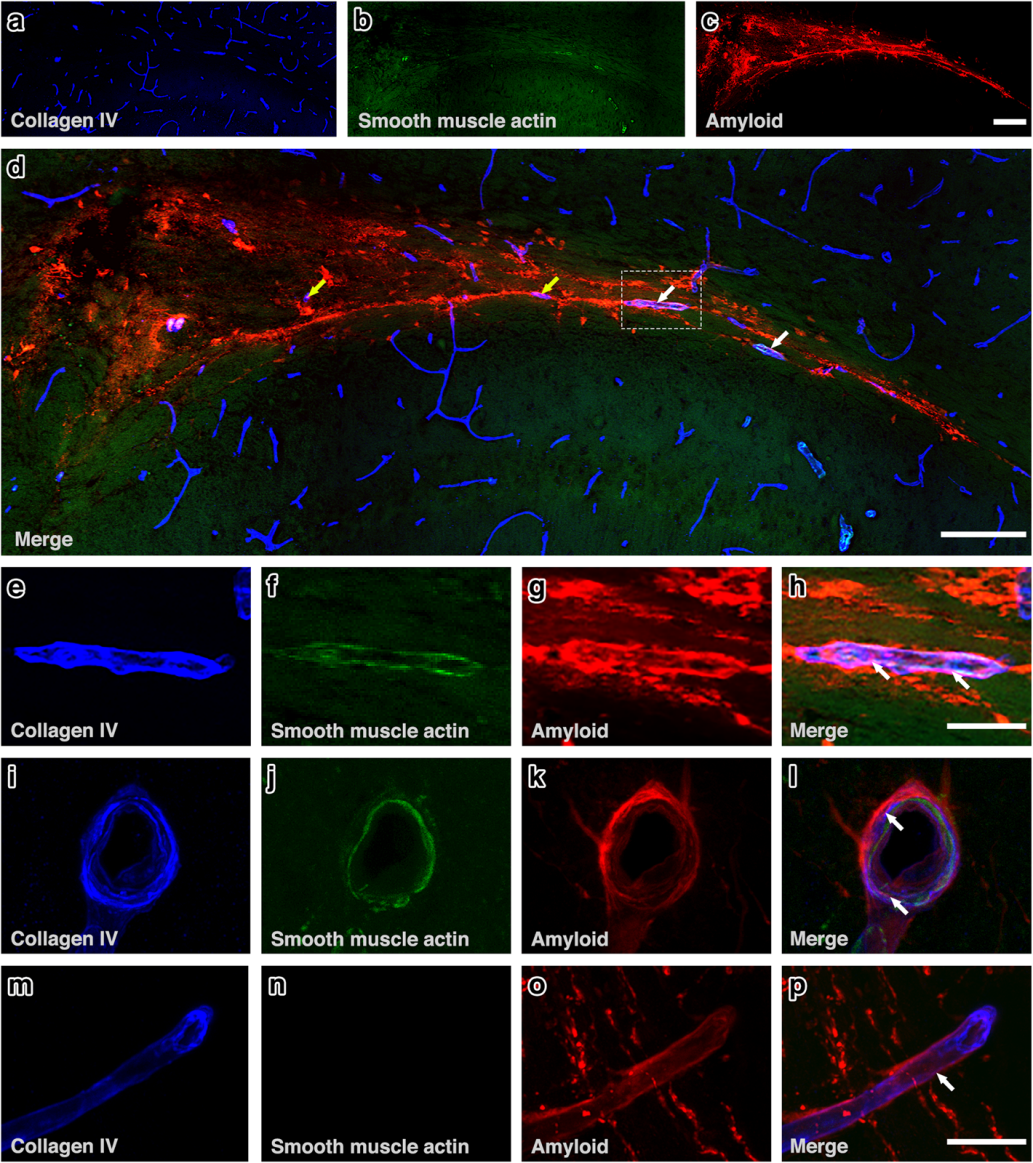
IPAD in corpus callosum. **(a-c)** Distribution of amyloid-β in relation to collagen IV and smooth muscle actin. Amyloid-β **(red)** was observed along the white matter tracts **(c & d)** and co-localising **(Pink)** with collagen IV in the walls of capillaries **(yellow arrows)** and arterioles **(white arrows)**. **(e-h)** An arteriole in the white matter - see box in **(d)** -showing Aβ in the tunica media. Merging of the blue collagen IV staining in the basement membranes in **(e)** with red amyloid **(g)** produces a pink colour of co-localisation in **(h)**. **(i-l)** A leptomeningeal artery in the hippocampal fissure abutting on to the white matter shows Aβ co-localized **(pink)** with collagen IV in the tunica media **(lower arrow)** and in the adventitia **(upper arrow)**. **(m-p)** shows red Aβ **(arrow)** in the wall of a white matter capillary that is also stained blue for collagen IV in the basement membrane. Scale bars **a-d** = 200 μm, **e – h =** 20 μm **& i –*p*** = 10 μm

Qualitative assessment revealed the pattern of IPAD normally observed in the hippocampus [30], characterised by colocalisation of fluorescent Aβ40 with collagen IV within the walls of arterioles and capillaries and few venules and in the parenchyma, mainly in the granule cell layer (Fig. 2). In the corpus callosum, fluorescent Aβ40 was observed mainly along the white matter tracts and colocalising with collagen IV within the walls of capillaries, some arterioles and few venules (Fig. 3).

**Fig. 4:**
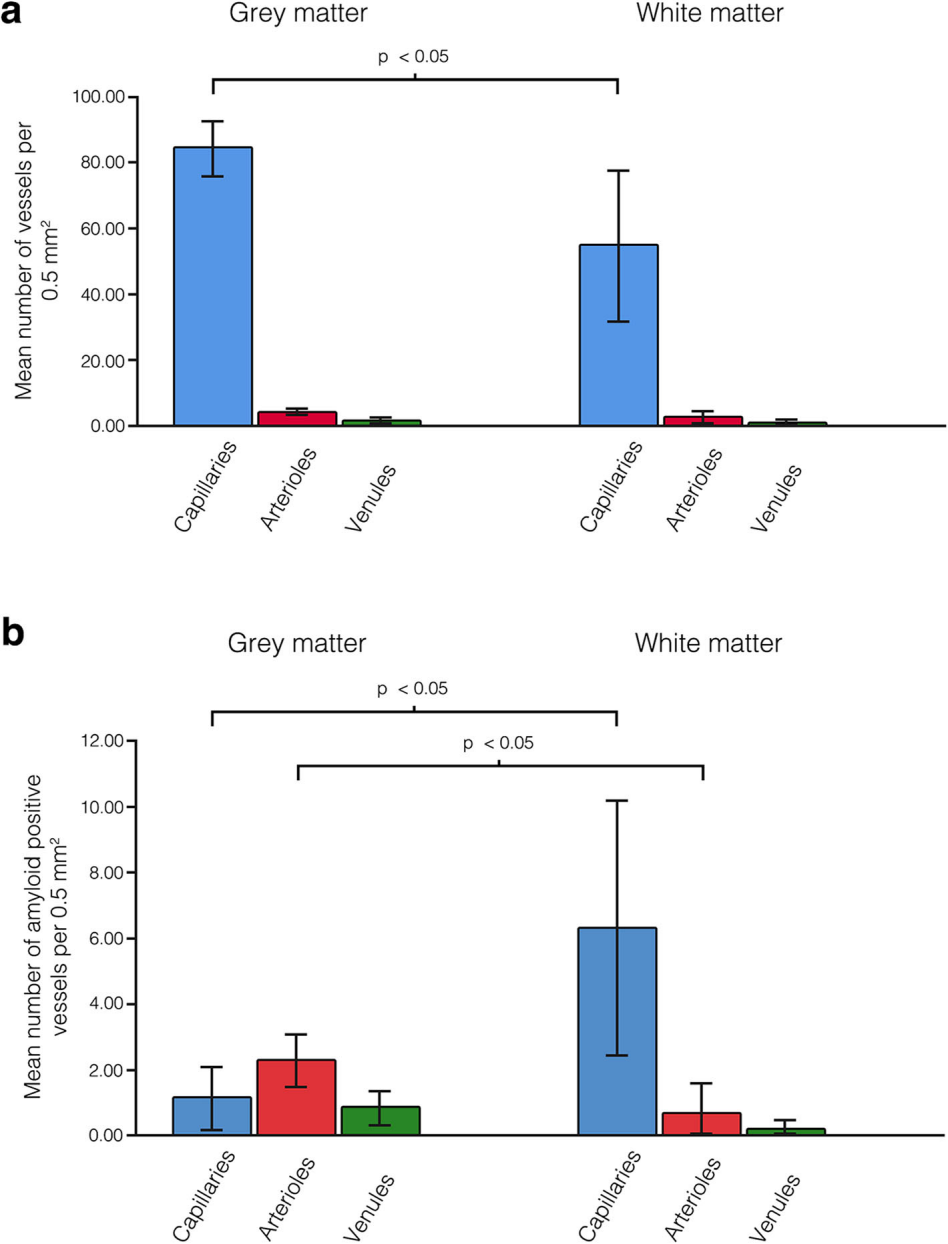
Comparison of vessel density **(a)** and the density of vessels with fluorescent Aβ in their walls **(b)** between the hippocampus (grey matter) and corpus callosum (white matter). The density of arterioles with fluorescent Aβ in their vessel walls was significantly higher in the hippocampus but the density of capillaries with fluorescent Aβ in their vessel walls significantly higher in the corpus callosum **(b)** despite the significant reduction in density of capillaries in the corpus callosum **(a).** Error bars: +/− 2 SE

Quantitative assessment of IPAD revealed significant differences in the density of capillaries and arterioles with Aβ40 in their vessel walls between the hippocampus and corpus callosum. The density of arterioles with Aβ40 in their vessel walls was significantly higher in the hippocampus versus corpus callosum (2.3 vs 0.6 per 0.5 mm^2^, *p* < 0.05). Conversely, the density of capillaries with Aβ40 in their vessel walls was significantly higher in the corpus callosum versus hippocampus (1.1 vs. 6.3 per 0.5 mm^2^, *p* < 0.05) (Fig. 4).

Previous work by Cavaglia et.al [31] showed regional variation in the density of cerebral capillaries. We observed similar variation with a significant reduction in the density of capillaries in corpus callosum compared to the hippocampus (84 vs 54.5 per 0.5mm^2^, *p* < 0.05) but we observed no significant differences in density of arterioles (4.4 vs. 2.3 per 0.5 mm^2^, *p* = 0.134) or venules (1.9 vs.1.1 per 0.5 mm^2,^*p* = 0.063) (Fig. 4).

### The expression of fibronectin and laminin is altered in human white matter hyperintensities

**Fig. 5:**
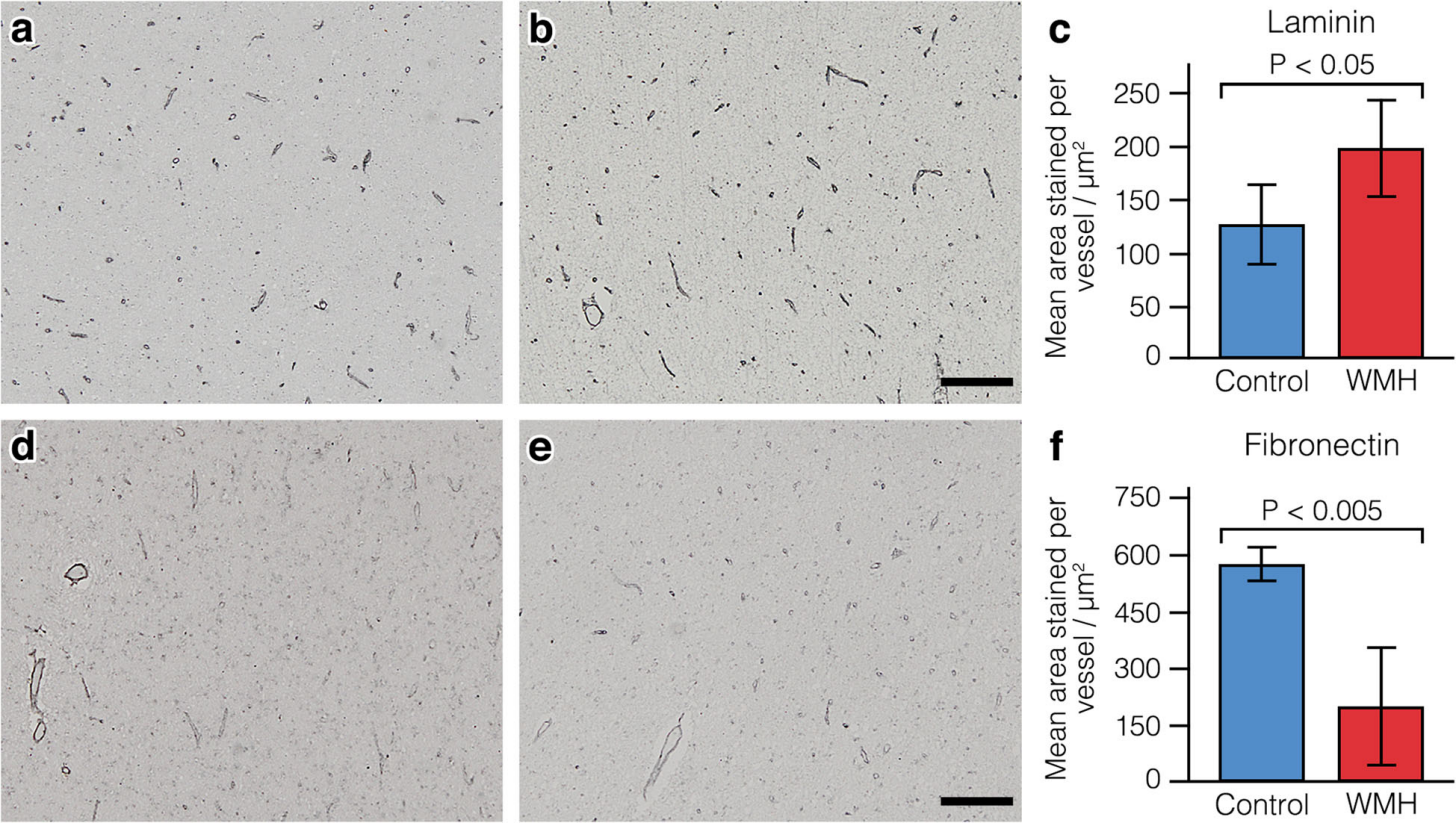
Laminin and fibronectin expression in the white matter in subjects with white matter hyperintensities and controls. **a** Representative image of laminin staining in the control group. **b** Representative image of laminin staining in the WMH group. **c** Histogram showing the mean area of laminin staining per vessel. **d** Representative image of fibronectin staining in the control group. **e** Representative image of fibronectin staining in the WMH group. **f** Histogram showing the mean area of fibronectin staining per vessel. Scale bars indicate 200um. Error bars indicate +/− 2 SE

Experimental work in rodents demonstrate that ischemia in the white matter is characterised by an upregulation of the extracellular matrix proteins laminin and fibronectin [24–26]. Previous work by our group also show that ageing and hypoperfusion lead to a changes in the distribution of laminin and fibronectin in the white matter [10, 32]. To ascertain if similar changes in the distribution occur in the white matter in WMH, we next used immunohistochemistry to assess changes in laminin and fibronectin expression in human brain slices of WMH and age matched control white matter. We observed a significant increase in laminin expression (197.81 μm^2^ per vessel vs 126.33 μm^2^ per vessel, *p* < 0.05) and a significant decrease in fibronectin expression (199.50 μm^2^ per vessel vs 574.22 μm^2^ per vessel, *p* < 0.005) in the white matter of subjects with WMH compared to aged-matched controls (Fig. 5).

### Expression of Fibronectin and Laminin by human vascular smooth muscle cells is altered with an increase in CO_2_

Previous mathematical modelling by our group showed that the vasomotion generated by cycles of contraction and relaxation of smooth muscle cells is vital for efficient IPAD [17, 18]. Loss of this function, as seen with ageing and in CAA, results in hypoperfusion, ischaemia and a failure of IPAD [17, 19, 20]. We therefore next investigated whether hypercapnia as a model of hypoperfusion/hypoxia, as in WMH, affects the function of smooth muscle cells. We exposed cultured HBVSMC to an increase in CO_2_ and assessed cell proliferation and metabolic activity and production of laminin and fibronectin.

**Fig. 6:**
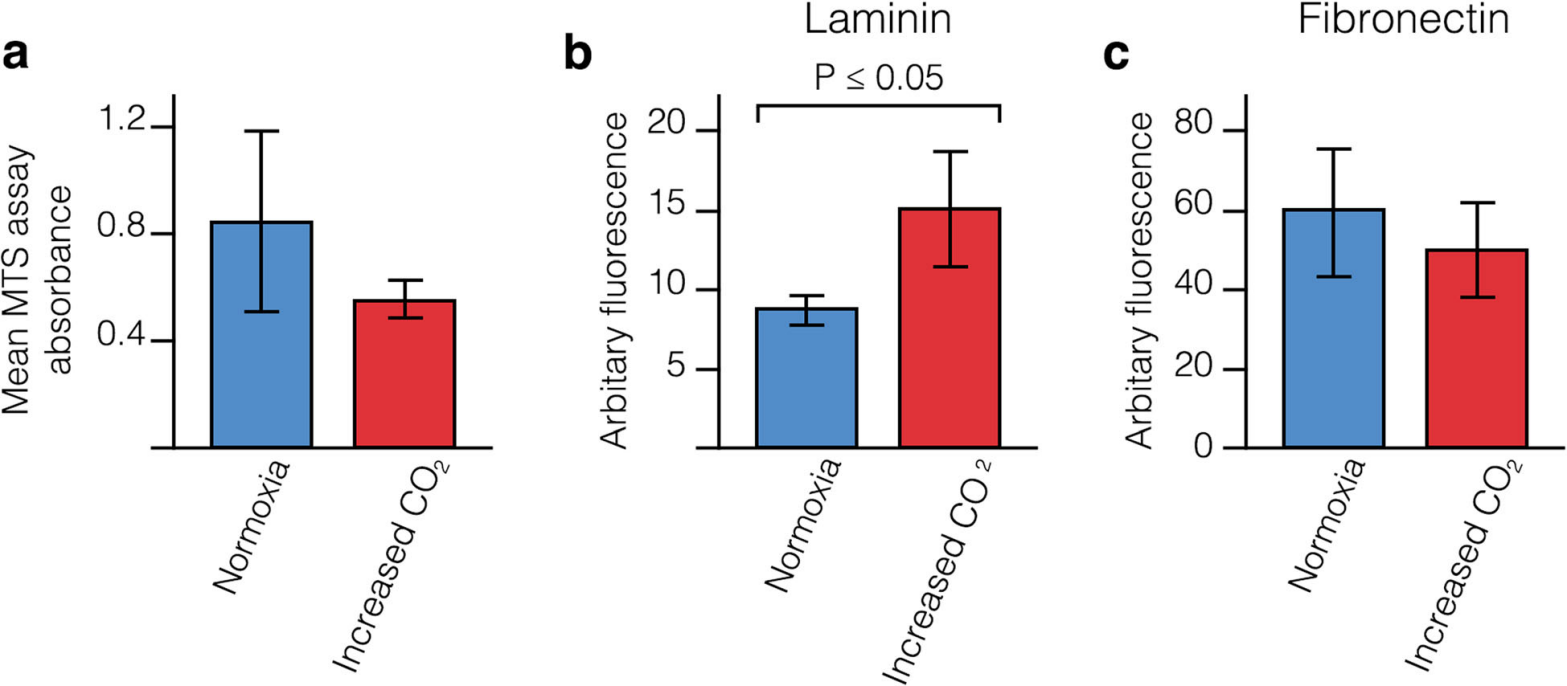
Mean MTS absorbance and mean fluorescence intensity of HBVSMC exposed to increased levels of CO_2_. **a** Mean MTS absorbance was reduced but not significantly.**b** Laminin staining was significantly higher in cultures exposed to increased levels of CO_2_ compared to the normoxia cultures (*p* ≤ 0.05). **c** There was a trend towards a decrease in the fluorescence intensity of fibronectin staining but this was not statistically significant. Error bars: +/− 1 SE

**Fig. 7:**
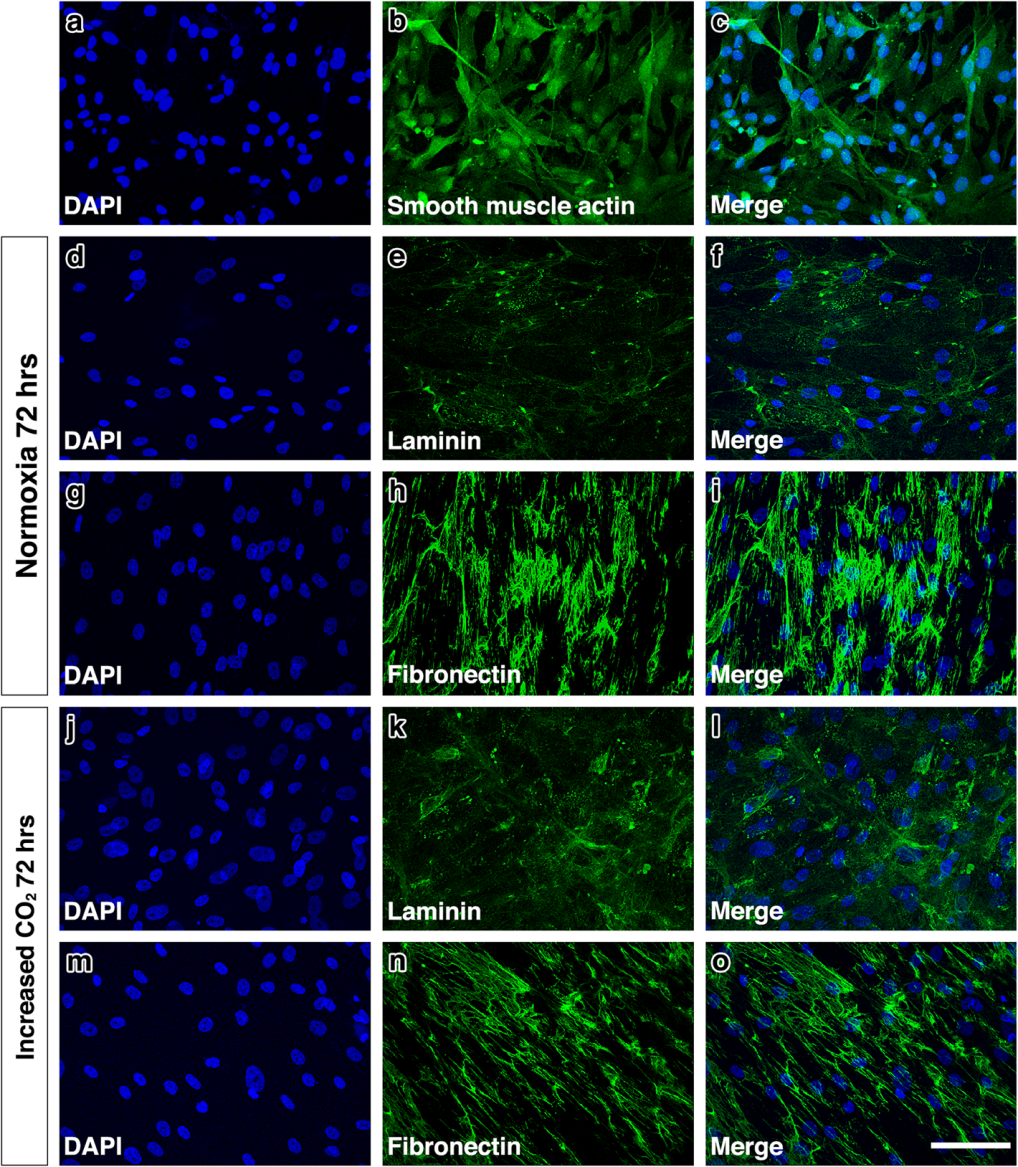
Laminin and fibronectin staining in HBVSMC cultures after 72 h exposure to normoxia and hypoxia. Reference images **(a – c)** show the presence of smooth muscle actin in the HBVSMCs used in this study. The pattern of laminin staining **(e,k)** was similar in the HBVSMC cultures but there was a significant increase in expression with increased levels of CO_2_**(l)** compared to normoxice cultures **(f)**. Fibronectin staining **(h,n)** had a web-like appearance in both cultures but staining appeared more intense in the normoxic cultures **(i)** compared to cultures exposed to increased levels of CO_2_**(o)**. Cell density, indicated by DAPI stained nuclei **(d, g, j, m)**, did not appear to be altered in normoxic conditions or conditions of increased levels of CO_2._ Scale bar = 50 μm

Following 72 h of exposure to increased levels of CO_2_, we observed a reduction in MTS absorbance of HBVSMC compared to normoxic conditions but this difference was not significant (0.851 vs. 0.560, *p* = 0.631) (Fig. 6). However, analysis by confocal microscopy and subsequent quantification of laminin and fibronectin expression, revealed a significant increase in expression of laminin in HBVSMC exposed to 8% CO_2_ in 92% air (p ≤ 0.05).. Fibronectin was decreased but not significantly (*p* = 0.827) (Figs. 6 & 7).

## Discussion

Of the two major functions of cerebral arteries discussed in this paper in relation to WMH, blood supply to the white matter has received more attention in the literature than elimination of fluid by IPAD. The results of the present study provide evidence that reduced capacity for IPAD from the white matter compared with grey matter together with a degree of hypoxia are involved in the aetiology of WMH.

### Reduced capacity for IPAD in the white matter compared with grey matter

We tested the hypothesis that the dynamics of IPAD in the cerebral white matter differ from those in the grey matter of the hippocampus. There are two major observations in our results that support this hypothesis. We found *First:* that, in rodents, the density of capillaries in the white matter is lower than in the grey matter of the hippocampus. As delivery of nutrients to the brain is via vascular capillaries, the lower density in the white matter suggests a lower capacity in white matter compared to grey matter for the delivery of oxygen and other nutrients. This may result in an increased risk of ischaemia/hypoxia in the white matter over grey matter in the presence of diseases such as arteriosclerosis and CAA in the arteries supplying the white matter; white matter appears to have a lower capacity for delivery of nutrients than grey matter. *Second*: the lower density of capillaries suggests that there is a lower capacity for IPAD in white matter when compared to grey matter. The reduced capacity of IPAD is shown in the current tracer experiments. When soluble Aβ was injected as a tracer into the white matter it was cleared more slowly from the capillaries compared to grey matter. A similar effect is seen with increasing age in the grey matter [10]. It seems therefore that the lower capacity of IPAD in the white matter may make it more vulnerable to failure when feeder arteries are affected by age-related changes and CAA that are both known to impede IPAD [4].

**Fig. 8:**
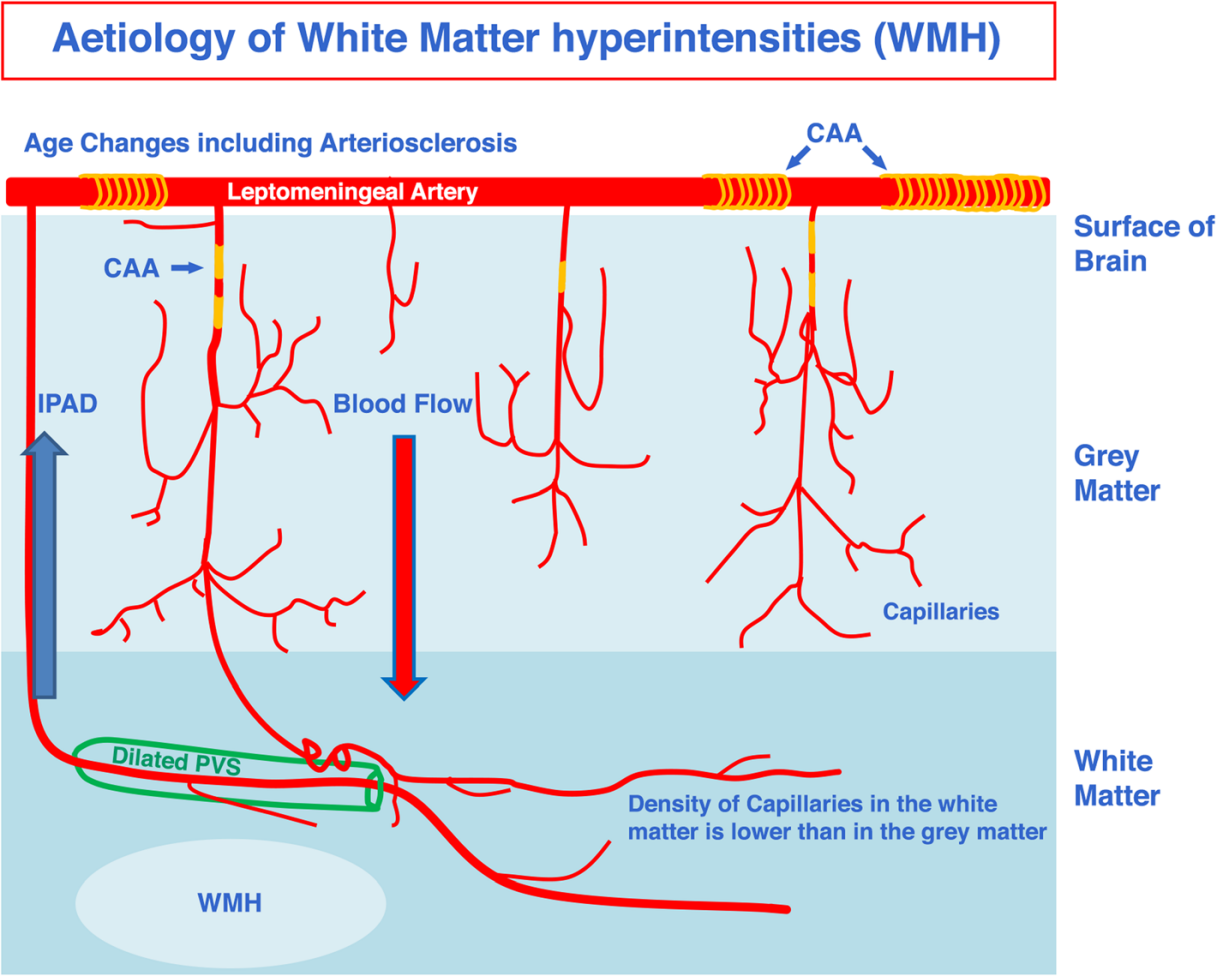
Aetiology of White Matter Hyperintensities (WMH) in the human brain. Two of the major functions of cerebral arteries, arterioles and capillaries are (**a**) Blood Flow and the supply of nutrients to brain tissues (Red Arrow) and (**b**) Elimination of Interstitial Fluid (ISF) and soluble metabolites from brain tissues along basement membranes within the walls of capillaries and arteries – IPAD (Intramural Peri-Arterial Drainage) (Blue Arrow). Age changes in arteries, such as arteriosclerosis, impair both blood flow and IPAD resulting in ischaemic/hypoxic changes and a reduced drainage of fluid, particularly from the white matter, leading to WMH. A lower density of capillaries in white matter also reduces the capacity for IPAD and may be a causal factor for WMH in the ageing brain. Reduced IPAD is associated with the deposition of Amyloid-β (Aβ) in the IPAD pathways of cortical and leptomeningeal arteries as Cerebral Amyloid Angiopathy (CAA). The capacity of IPAD is further reduced by CAA and is associated with dilated, fluid-containing perivascular spaces (PVS) around arteries in the white matter and with WMH. Diagram adapted from [37]

In the normal white matter of humans, the total number of capillaries is at least 49% lower compared to the grey matter in humans [33]. We demonstrate a similar reduction in the number of capillaries in the rodent white matter compared to the grey matter. In subjects with WMH, the capillary density decreases further, along with thickening of the capillary wall, leading to hypoperfusion [34]. The ISF entering the capillary bed in the white matter drains towards the coiled, tortuous arterioles situated in the white matter and then into the cortical arterioles [35, 36]. Figure 8 shows the relationship between arteries in cerebral cortex to those in the underlying white matter in the human brain [37]. Most of the blood for the subcortical white matter is supplied by arteries that branch from the leptomeningeal arteries and traverse the cortex, with or without branching, to supply the underlying white matter. Figure 8 also suggests how both blood flow and IPAD could be reduced by the development of arteriosclerosis and CAA in the leptomeningeal and cortical arteries.

There are other pathologies in the white matter that may also reflect a reduced capacity for IPAD in the white matter, supporting the notion that in vivo imaging indicates that water shifts are prominent and represent early changes in WMH [3]. Vasogenic oedema that results from tumours in the brain or subarachnoid haemorrhage mainly involves the white matter [38, 39]. This sugests that fluid dynamics in the white matter may differ from those in the grey matter. In the present study it has been shown that the density of capillaries is lower in the white matter than in grey matter whereas the densities of arterioles is similar. As capillary basement membranes are the entry portals for IPAD by which ISF and solutes drain from brain tissue this relative shortage of capillaries in the white matter may be a factor in the reduced capacity for IPAD. Similarly observations in acute hydrocepahlus support the conclusion that the capacity of IPAD may be lower in white matter than in grey matter. Obstruction of CSF drainage from the cerebral ventricles results in dilatation of the ventricular system and the accumulation of fluid in the periventricular white matter in the acute stages of hydrocephalus with the slowly progressive destruction of white matter fibres and gliosis [40]. The grey matter is only affected to a minor degree by the accumulation of fluid at its junction with the white matter even when the white matter is very severely damaged [41]. These observations support the concept that the capacity of IPAD to eliminate fluid is lower in the white matter than in the grey matter. Our experimental studies focused only on the pattern of IPAD in the corpus callosum of mice, as it is the only part of the white matter that is large enough for intracerebral injections. There may be differences in the pattern of IPAD from the different parts of the white matter (for example subcortical or periventricular), as there are differences in the grey matter reflected also in different patterns of CAA [32, 42].

Our results in post-mortem brains indicate that fibronectin decreases in WMH. The levels of fibronectin were not changed in human cerebral smooth muscle cells subjected to hypercapnia as a model of hypoxic hypoperfusion. This is in contrast to the results obtained in experimental work with rodents, suggesting that human VSMCs react to hypoxia by a reduction in the synthesis of fibronectin, in contrast to rodent VSMCs. Hypoxia in the brain results in an increase matrix metalloproteinase-2 (MMP2) and there is a complex relationship between fibronectin and MMP2 in humans that varies according to the changes in the time from the hypoxic event [43, 44]. Our results in the human brain tissue reflect changes in chronic hypoxia which are considerably different to the experimental hypercapnia in smooth muscle cell lines. As fibronectin is a substrate for α5β1 integrin receptor [2, 45, 46] and both are involved in axonal regeneration, our results support the hypothesis that axonal regeneration is impaired in WMH.

It has been demonstrated in experimental work in rodents that laminin increases after hypoxia [26, 47], but the results in human brains are controversial. Our own results in post-mortem human cortical occipital sections demonstrate that there was no significant difference in the percentage area of collagen IV, laminin or fibronectin in CAA compared to age matched control sections [48]. However, collagen IV, a key glycoprotein component of the BM has been recently shown as increased in human WMH [49]. It may be that the hypoxic environment leads to an increase in laminin synthesis as a compensatory mechanism for the change in homeostasis of the brain, possibly leading to angiogenesis [50].

It has been known for several decades that the majority of the interstitial fluid in the white matter drains along the white matter tracts [51, 52]. In addition to the bulk flow along white matter tracts our study demonstrates that IPAD of soluble Aβ40 is also present in the white matter and occurs preferentially along the walls of capillaries whereas in the grey matter it occurs preferentially along the walls of arteries. The change in pattern of laminin and fibronectin expression in the white matter in WMH does correlate with the changes seen with hypoxia in cultures of VSMCs. However this does not necessarily define the reason for the hypoxia in WMH. The question still remains as to whether the hypoxia is due to poor blood supply or is an effect of the increased fluid in the extracellular spaces separating tissue elements from their oxygen supply in the blood.

Many of the fine details of fluid dynamics withn the brain tissue and IPAD await explanation but what seems certain is the involvement of arteries and capillaries not only in the delivery of blood but also in the drainage of fluid and soluble matabolites from the brain. It may be possible therefore to combine therpeutic approaches to resolving ischaemia/hypoxia and failure of IPAD in WMH.

## Data Availability

The datasets used and/or analysed during the current study are available from the corresponding author on reasonable request.

## References

[1] Grinberg LT, Thal DR (2010). Vascular pathology in the aged human brain. Acta Neuropathol.

[2] McAleese KE, Walker L, Graham S, Moya ELJ, Johnson M, Erskine D (2017). Parietal white matter lesions in Alzheimer’s disease are associated with cortical neurodegenerative pathology, but not with small vessel disease. Acta Neuropathol.

[3] Wardlaw JM, Valdes Hernandez MC, Munoz-Maniega S (2015). What are White Matter Hyperintensities Made of? Relevance to Vascular Cognitive Impairment. J Am Heart Assoc.

[4] Weller RO, Hawkes CA, Kalaria RN, Werring DJ, Carare RO (2015). White matter changes in dementia: role of impaired drainage of interstitial fluid. Brain Pathol.

[5] Szentistvanyi I, Patlak CS, Ellis RA, Cserr HF (1984). Drainage of interstitial fluid from different regions of rat brain. AmJ Physiol.

[6] McIntee FL, Giannoni P, Blais S, Sommer G, Neubert TA, Rostagno A (2016). In vivo differential brain clearance and catabolism of monomeric and Oligomeric Alzheimer’s Abeta protein. Front Aging Neurosci.

[7] Carare RO, Bernardes-Silva M, Newman TA, Page AM, Nicoll JA, Perry VH (2008). Solutes, but not cells, drain from the brain parenchyma along basement membranes of capillaries and arteries: significance for cerebral amyloid angiopathy and neuroimmunology. NeuropatholApplNeurobiol..

[8] Albargothy NJ, Johnston DA, MacGregor-Sharp M, Weller RO, Verma A, Hawkes CA et al (2018) Convective influx/glymphatic system: tracers injected into the CSF enter and leave the brain along separate periarterial basement membrane pathways. Acta Neuropathol 136(1):139–152 10.1007/s00401-018-1862-7 PMC6015107 29754206

[9] Tarasoff-Conway JM, Carare RO, Osorio RS, Glodzik L, Butler T, Fieremans E (2015). Clearance systems in the brain-implications for Alzheimer disease. Nat Rev Neurol.

[10] Hawkes CA, Hartig W, Kacza J, Schliebs R, Weller RO, Nicoll JA (2011). Perivascular drainage of solutes is impaired in the ageing mouse brain and in the presence of cerebral amyloid angiopathy. Acta Neuropathol.

[11] Carare RO, Hawkes CA, Jeffrey M, Kalaria RN, Weller RO (2013). Review: cerebral amyloid angiopathy, prion angiopathy, CADASIL and the spectrum of protein elimination failure angiopathies (PEFA) in neurodegenerative disease with a focus on therapy. Neuropathol Appl Neurobiol.

[12] Weller RO, Hawkes CA, Carare RO, Hardy J (2015). Does the difference between PART and Alzheimer’s disease lie in the age-related changes in cerebral arteries that trigger the accumulation of Abeta and propagation of tau?. Acta Neuropathol.

[13] Engelhardt B, Carare RO, Bechmann I, Flugel A, Laman JD, Weller RO (2016). Vascular, glial, and lymphatic immune gateways of the central nervous system. Acta Neuropathol.

[14] Engelhardt B, Vajkoczy P, Weller RO (2017). The movers and shapers in immune privilege of the CNS. Nat Immunol.

[15] Weller RO, Djuanda E, Yow HY, Carare RO (2009). Lymphatic drainage of the brain and the pathophysiology of neurological disease. Acta Neuropathol.

[16] Diem AK, MacGregor Sharp M, Gatherer M, Bressloff NW, Carare RO, Richardson G (2017). Arterial pulsations cannot drive intramural Periarterial drainage: significance for Abeta drainage. Front Neurosci.

[17] Aldea R, Weller RO, Wilcock DM, Carare RO, Richardson G (2019). Cerebrovascular smooth muscle cells as the drivers of intramural Periarterial drainage of the brain. Front Aging Neurosci.

[18] van Veluw SJ, Hou SS, Calvo-Rodriguez M, Arbel-Ornath M, Snyder AC, Frosch MP (2020). Vasomotion as a driving force for Paravascular clearance in the awake mouse brain. Neuron..

[19] Okamoto Y, Yamamoto T, Kalaria RN, Senzaki H, Maki T, Hase Y (2012). Cerebral hypoperfusion accelerates cerebral amyloid angiopathy and promotes cortical microinfarcts. Acta Neuropathol.

[20] Barker R, Wellington D, Esiri MM, Love S (2013). Assessing white matter ischemic damage in dementia patients by measurement of myelin proteins. J Cereb Blood Flow Metab.

[21] Hallmann R, Horn N, Selg M, Wendler O, Pausch F, Sorokin LM (2005). Expression and function of laminins in the embryonic and mature vasculature. Physiol Rev.

[22] Horsburgh K, Wardlaw JM, van Agtmael T, Allan SM, Ashford MLJ, Bath PM (2018). Small vessels, dementia and chronic diseases - molecular mechanisms and pathophysiology. Clin Sci (Lond).

[23] Joutel A, Haddad I, Ratelade J, Nelson MT (2016). Perturbations of the cerebrovascular matrisome: a convergent mechanism in small vessel disease of the brain?. J Cereb Blood Flow Metab.

[24] Halder SK, Kant R, Milner R (2018). Chronic mild hypoxia promotes profound vascular remodeling in spinal cord blood vessels, preferentially in white matter, via an alpha5beta1 integrin-mediated mechanism. Angiogenesis..

[25] Halder SK, Kant R, Milner R (1700). Chronic mild hypoxia increases expression of laminins 111 and 411 and the laminin receptor alpha6beta1 integrin at the blood-brain barrier. Brain Res.

[26] Hawkes CA, Michalski D, Anders R, Nissel S, Grosche J, Bechmann I (2013). Stroke-induced opposite and age-dependent changes of vessel-associated markers in co-morbid transgenic mice with Alzheimer-like alterations. Exp Neurol.

[27] Fernando MS, O’Brien JT, Perry RH, English P, Forster G, McMeekin W (2004). Comparison of the pathology of cerebral white matter with post-mortem magnetic resonance imaging (MRI) in the elderly brain. Neuropathol Appl Neurobiol.

[28] Wharton SB, Simpson JE, Brayne C, Ince PG (2015). Age-associated white Matter lesions: the MRC cognitive function and ageing study. Brain Pathol.

[29] Rueden CT, Schindelin J, Hiner MC, DeZonia BE, Walter AE, Arena ET (2017). ImageJ2: ImageJ for the next generation of scientific image data. BMC Bioinformatics.

[30] Morris AW, Sharp MM, Albargothy NJ, Fernandes R, Hawkes CA, Verma A (2016). Vascular basement membranes as pathways for the passage of fluid into and out of the brain. Acta Neuropathol.

[31] Cserr HF, Ostrach LH (1974). Bulk flow of interstitial fluid after intracranial injection of blue dextran 2000. Exp Neurol.

[32] Hawkes CA, Gatherer M, Sharp MM, Dorr A, Yuen HM, Kalaria R (2013). Regional differences in the morphological and functional effects of aging on cerebral basement membranes and perivascular drainage of amyloid-beta from the mouse brain. Aging Cell.

[33] Hase Y, Ding R, Harrison G, Hawthorne E, King A, Gettings S (2019). White matter capillaries in vascular and neurodegenerative dementias. Acta Neuropathol Commun.

[34] Mozumder M, Pozo JM, Coelho S, Costantini M, Simpson J, Highley JR (2019). Quantitative histomorphometry of capillary microstructure in deep white matter. NeuroImage Clinical.

[35] Nonaka H, Akima M, Hatori T, Nagayama T, Zhang Z, Ihara F (2003). The microvasculature of the cerebral white matter: arteries of the subcortical white matter. J Neuropathol Exp Neurol.

[36] Nonaka H, Akima M, Hatori T, Nagayama T, Zhang Z, Ihara F (2003). Microvasculature of the human cerebral white matter: arteries of the deep white matter. Neuropathology.

[37] Duvernoy HM, Delon S, Vannson JL (1981). Cortical blood vessels of the human brain. Brain Res Bull.

[38] Tiller-Borcich JK, Fike JR, Phillips TL, Davis RL (1987). Pathology of delayed radiation brain damage: an experimental canine model. Radiat Res.

[39] Weimer JM, Jones SE, Frontera JA (2017). Acute cytotoxic and Vasogenic edema after subarachnoid hemorrhage: a quantitative MRI study. AJNR Am J Neuroradiol.

[40] Weller RO, Wisniewski H, Shulman K, Terry RD (1971). Experimental hydrocephalus in young dogs: histological and ultrastructural study of the brain tissue damage 142. J NeuropatholExpNeurol.

[41] Weller RO, Wisniewski H (1969). Histological and ultrastructural changes with experimental hydrocephalus in adult rabbits 151. Brain.

[42] Attems J, Jellinger K, Thal DR, Van Nostrand W (2011). Review: sporadic cerebral amyloid angiopathy. Neuropathol Appl Neurobiol.

[43] Steffensen B, Xu X, Martin PA, Zardeneta G (2002). Human fibronectin and MMP-2 collagen binding domains compete for collagen binding sites and modify cellular activation of MMP-2. Matrix Biol.

[44] Hua Y, Zhang W, Xie Z, Xu N, Lu Y (2016). MMP-2 is mainly expressed in arterioles and contributes to cerebral vascular remodeling associated with TGF-beta1 signaling. J Mol Neurosci.

[45] Matter ML, Zhang Z, Nordstedt C, Ruoslahti E (1998). The alpha5beta1 integrin mediates elimination of amyloid-beta peptide and protects against apoptosis. J Cell Biol.

[46] Tom VJ, Doller CM, Malouf AT, Silver J (2004). Astrocyte-associated fibronectin is critical for axonal regeneration in adult white matter. J Neurosci.

[47] Yao Y (2019). Basement membrane and stroke. J Cerebral Blood Flow Metab.

[48] Keable A, Fenna K, Yuen HM, Johnston DA, Smyth NR, Smith C et al (2015) Deposition of amyloid beta in the walls of human leptomeningeal arteries in relation to perivascular drainage pathways in cerebral amyloid angiopathy. Biochim Biophys Acta 1862(5):1037–1046 10.1016/j.bbadis.2015.08.024 PMC4827375 26327684

[49] Waller R, Baxter L, Fillingham DJ, Coelho S, Pozo JM, Mozumder M (2019). Iba-1−/CD68+ microglia are a prominent feature of age-associated deep subcortical white matter lesions. PLoS One.

[50] Ji K, Tsirka SE (2012). Inflammation modulates expression of laminin in the central nervous system following ischemic injury. J Neuroinflammation.

[51] Geer CP, Grossman SA (1997). Interstitial fluid flow along white matter tracts: a potentially important mechanism for the dissemination of primary brain tumors. J Neuro-Oncol.

[52] Cserr HF, DePasquale M, Patlak CS, Pullen RG (1986). Convection of cerebral interstitial fluid and its role in brain volume regulation 28. AnnNYAcadSci..

